# Nutritional and Health Potential of Edible Seeds: Micronutrient Bioavailability and Mechanistic Insights

**DOI:** 10.1002/fsn3.71480

**Published:** 2026-01-28

**Authors:** Nighat Raza, Alina Sadaf, Reeha Mushtaq, Mirza Abid Mehmood, Luqman Amrao, Fakhar Islam, Muhammad Afzaal, Mohd Asif Shah

**Affiliations:** ^1^ Deparment of Food Science and Technology Muhammad Nawaz Shareef University of Agriculture Multan Pakistan; ^2^ Department of Dietetics and Nutrition Lahore General Hospital Lahore Pakistan; ^3^ Institute of Plant Protection Muhammad Nawaz Shareef University of Agriculture Multan Pakistan; ^4^ Department of Plant Pathology University of Agriculture Faisalabad Pakistan; ^5^ Department of Food Science Government College University Faisalabad Pakistan; ^6^ Department of Food Science and Technology Nur International University Lahore Pakistan; ^7^ Department of Agriculture Economics Kardan University Kabul Afghanistan; ^8^ Division of Research and Development Lovely Professional University Phagwara Punjab India; ^9^ University Centre for Research and Development Chandigarh University Mohali Punjab India

**Keywords:** bioactive constituents, biochemical mechanism, edible seeds, micronutrients

## Abstract

Edible seeds have gained substantial scientific attention for their exceptional nutrient density and potential health‐promoting properties. They are rich in dietary fiber, high‐quality proteins, mono‐ and polyunsaturated fatty acids, omega‐3 fatty acids, vitamins (E, C, and K), and minerals such as magnesium, zinc, potassium, and iron. Bioactive compounds like polyphenols, carotenoids, and peptides contribute to their strong antioxidant and anti‐inflammatory effects, helping to mitigate oxidative stress and inflammation linked to chronic diseases. This review focuses on pumpkin, flax, sesame, chia, and melon seeds, valuable sources of essential micronutrients and bioactives with demonstrated nutraceutical potential. Pumpkin seeds enhance immune strength because of their mineral profile, whereas chia seeds provide omega‐3 fatty acids associated with neuroprotection and anti‐Alzheimer's effects. The omega‐3 content of flax and chia seeds offers cardioprotective benefits, whereas sesame lignans (sesamin) exhibit lipid‐lowering and anti‐aging properties. Flaxseed's secoisolariciresinol diglycoside (SDG) contributes to cardiovascular and anti‐cancer effects, and melon seed squalene supports immune health and exerts anti‐cancer activity. Mechanistic studies highlight these seeds' ability to regulate molecular pathways related to oxidative stress, inflammation, hypertension, diabetes, cancer, and metabolic disorders. Their bioactive constituents act through antioxidant, anti‐inflammatory, and metabolic‐regulating mechanisms, validating their classification as functional foods. Evidence from clinical and biochemical studies largely supports these benefits, although some claims stem from preliminary or in vitro findings. Overall, pumpkin, flax, sesame, chia, and melon seeds demonstrate significant potential as natural sources of nutrients and bioactive compounds that promote cardiovascular, metabolic, and immune health. Their integration into daily diets and functional food formulations could play a vital role in preventing lifestyle‐related chronic diseases and enhancing overall well‐being.

## Graphical abstract

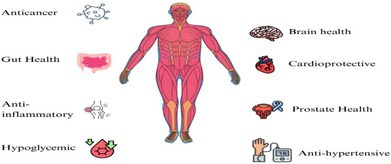

**Abbreviations**
ACE: angiotensin‐converting enzyme
ALA: alpha‐linolenic acid
AR: androgen receptor
BMI: body mass index
CRP: C‐reactive protein
DNA: deoxyribonucleic acid
ER: estrogen receptor
GLP‐1: glucagon‐like peptide 1
HbA1c: hemoglobin A1c
HDL: high‐density lipoprotein
IL‐6: interleukin 6
LDL: low‐density lipoprotein
LXR: liver X receptor
MAPK: mitogen‐activated protein kinase
MPO: myeloperoxidase
mTOR: mammalian target of rapamycin
NADPH: nicotinamide adenine dinucleotide phosphate
NF‐kB: nuclear factor kappa B
NOS: nitric oxide synthase
PI3K: phosphoinositide 3‐kinase
PUFA: polyunsaturated fatty acid
RNA: ribonucleic acid
ROS: reactive oxygen species
SCFA: short chain fatty acids
TG: triglycerides
TNF‐alpha: tumor necrosis factor alpha
VEGF: vascular endothelial growth factor
VLDL: very low‐density lipoprotein

## 1 Introduction

A healthy lifestyle is achieved if the population consumes a diet that is secure, healthy, and dense in nutrients, which can fulfill the needs of the human body for good health and well‐governed physical status. The demand for functional and nutritional food products that are capable of delivering health needs is growing because of the demand for a healthy lifestyle (Bhardwaj et al. 2020). For healthy maintenance and regulation of normal health, various micronutrients must be available in the body that can be consumed through a balanced diet. Bioactive nutritional compounds are present in the food composition that are required by the human body to function normally. These include protein, carbohydrates, fats, minerals (like calcium, zinc, magnesium, potassium), and vitamins (like vitamin C, E, and K), of which vitamins and minerals are micronutrients. Vitamins are organic compounds, such as fat‐soluble vitamins, which include vitamin A, D, E, and K. Water‐soluble vitamins are vitamin C and vitamin B. On the contrary, minerals are inorganic substances, such as macro minerals, if needed in excess of 100 g per day, such as calcium, magnesium, sodium, potassium, and chloride, whereas microminerals are needed in less than 100 g per day, such as iron, zinc, selenium, and iodine (Morris and Mohiuddin 2020).

It can be possible that the suggestion of daily consumption of these micronutrients has nothing to do with the expenditure of energy in physical activity (Bielik and Kolisek 2021). Throughout the lifetime, to keep health and well‐being intact, macronutrient and micronutrient consumption is essential, particularly in more susceptible groups such as children, pregnant women, and elderly people. Adequacy of these nutrients is needed in reproductive age to prevent negative effects (Morris and Mohiuddin 2020).

**FIGURE 1:**
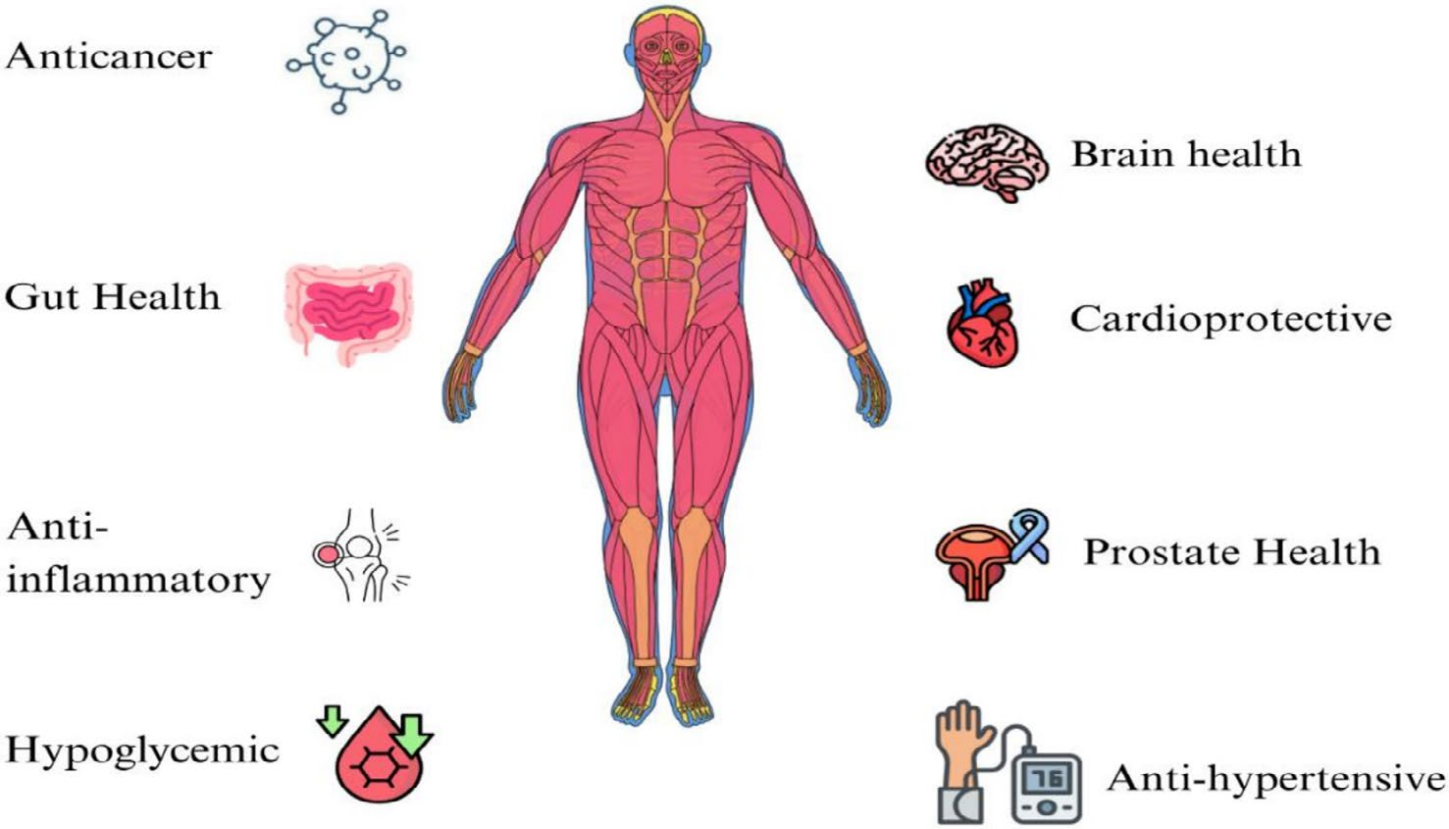
Nutraceutical impacts of seeds on health.

Micronutrient supplementation is not justified for all populations but rather for specific subpopulations. Food fortification is a potentially effective approach because it provides necessary micronutrients, but it cannot replicate the nutritional profile and health advantages found in naturally nutrient‐rich foods (Kakkar et al. 2023). By contrast, introducing functional ingredients as synergistic nutrients in minimally processed products can yield extra benefits beyond those achieved through fortification. Moreover, whereas some foods promote various positive health outcomes, ultra‐processed, calorie‐rich foods are linked to a range of non‐communicable diseases and health risks. Ultra‐processed foods, being highly palatable, tend to be widely consumed; when they constitute a significant part of the diet, they contribute to obesity (Beal and Ortenzi 2022). The data collected from Asia, China, and Korea show that the increased consumption of fast food leads to obesity and serious health concerns (Wu et al. 2021). A schematic diagram of nutraceutical health benefits of edible seeds is shown in Figure 1.

Increased blood levels of LDL can be a risk factor for heart disease, and are frequently linked to saturated fats. The increased level of SFA may be undesirable in terms of cardiovascular health. Both omega‐6 and omega‐3 fatty acids, which are vital lipids, are polyunsaturated fats (Senila et al. 2025). Saturated fatty acids (SFAs), which have no double bonds; monounsaturated fatty acids (MUFAs), which have one double bond; and polyunsaturated fatty acids (PUFAs), which have two or up to six double bonds, are the three types of FAs that make up lipids. The α‐linolenic acid (C18:3, (n‐3)) and linoleic acid (C18:2, (n‐6)) are the two main PUFA groups. PUFAs help stabilize protein and mineral deficiencies in the human body and lower insulin levels in diabetics. Edible seeds and mushrooms are the primary sources of unsaturated fats (Senila et al. 2020; Senila et al. 2024).

**FIGURE 2:**
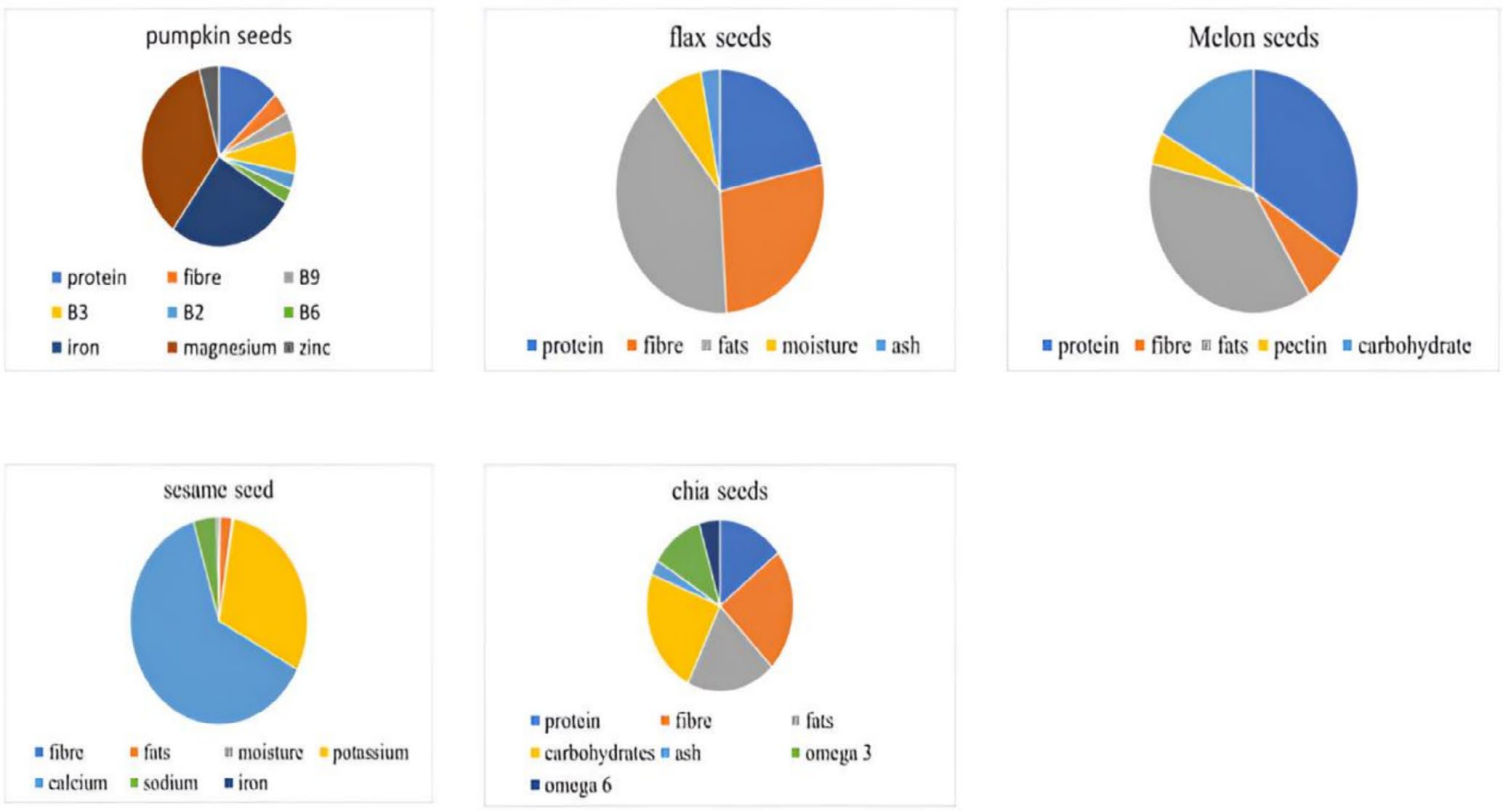
Nutrients per 100 g (Abbas et al. 2022; Capitani et al. 2012; Duarte et al. 2025; Ghafoor et al. 2018; Wei et al. 2022).

Seeds have been widely used to fight against diseases such as obesity. Therefore, diet‐related metabolic disorders, inflammation, body fat accumulation, and dyslipidemia increase the possibility of impaired insulin secretion, which results in cancer, obesity, and cardiovascular diseases (Moreno‐Valdespino et al. 2020). The fatty acids, particularly DHA, are of great importance to brain health as they play a role in the formation of the neural tube during pregnancy, a key component to create myelin, the insulation that envelops the nerve fibers and helps facilitate nerve impulses. These are implicated in neurogenesis and brain development. The mechanism to develop the neural tube that starts during pregnancy continues on until infancy, and it includes the cell division that begins in the neural tube after 7 weeks of pregnancy to form neurons and glial cells. Next, the nerve cells migrate to the brain, and axons and dendrites form from there. These are the ones that have branches that connect cells, allowing them to make synapses so that the signals can move. The nutrients, such as copper, vitamin A, and folic acid, affect the formation of the neural tube. The nutrients that have significant roles in the brain development processes include zinc, iron, fatty acids, choline, B vitamins, and iodine. These are involved in the formation of the neural tube, neurogenesis, and consist of enzymes that assist in cell division, the synthesis of DNA, and the proliferation of stem cells (Prado and Dewey 2014). The major nutrients of pumpkin, flax, melon, sesame, and chia seeds are shown in Figure 2.

Optimal health maintenance in the recent trends of society is a complex undertaking. Epidemiological evidence shows that the high prevalence of lifestyle‐linked conditions is due to the worsening of the dietary habits of contemporary societies and a lack of ability to cope with stress efficiently (Kakkar et al. 2023). The unhealthy pattern has been associated with a range of health complications, including chronic non‐communicable diseases that could be fatal (Al‐Jawaldeh and Abbass 2022). Therefore, there is a necessity to implement preventative and therapeutic methods for these ailments that are sustainable. People with lifestyle‐related diseases are advised to follow a well‐balanced diet, which also takes into account bioactive foods such as fruits, vegetables, nuts, seeds, herbs, spices, and soy foods (Kakkar et al. 2023).

This review explores the health potential of edible seeds, including sesame, flax, and pumpkin seeds. It aims to examine the bioavailability of key micronutrients in these seeds, clarify their health benefits, and explain the mechanisms behind these advantages. These seeds contain rich bioactive substances that contribute to various health‐promoting activities, such as anti‐inflammatory, antioxidant, antihypertensive, and metabolic‐regulating effects (Nunes et al. 2025). The review seeks to understand how these constituents are absorbed and utilized systemically, as well as the molecular mechanisms through which they act to prevent or treat chronic diseases like diabetes, cardiovascular diseases, and inflammatory conditions (Abbas et al. 2022). The need for plant‐based proteins, interest in functional foods, and growing health consciousness are all contributing factors to the growing trend of edible seed consumption worldwide. In the culinary, supplement, cosmetic, and nutraceutical industries, functional seeds, including chia, flaxseed, hemp, and nigella, are becoming increasingly popular. Although North America still has the largest market share, the Asia‐Pacific area is developing at the quickest rate because of urbanization and rising demand for plant‐based, high‐protein, and natural goods (News 2025).

## 2 Health Impacts of Bioactive Compounds and the Mechanisms of Action

### 2.1 Flaxseeds

*Linum usitatissimum L*. is the scientific name of linseed or flax that belongs to the Linaceae family, the tribe Lineae, and the order Malpighiales. Rapeseed mustard is the second largest produced rabi oilseed crop in India (after rapeseed mustard) and a major oilseed crop in terms of total seed production. Although there are about 230 species in the genus *Linum*, the only economically important species is linseed or flax‐cultivated linseed (Tadesse et al. 2010). In the complete flaxseed, there exists a true hull or testa, as well as a very thin endosperm; there are two embryos, and there are also the two embryo axes, and these make the flaxseed flat and shaped like an oval with a point on each end (Guan et al. 2021). Flaxseed contains lignans, omega‐3 fatty acids, dietary fiber, vitamins, minerals, and many other important elements that make it a superfood or naturally occurring food that has various bioactive components and health‐boosting properties (Gupta and Mishra 2021). Consumption of flaxseed can be a very significant element in the prevention of diseases, especially those that are caused by an unhealthy diet (Nowak and Jeziorek 2023).

Flaxseed contains a large quantity of dietary fiber (40%), soluble fiber contributing 25% and insoluble fiber contributing 75% (Singh et al. 2011). In an attempt to prevent diabetes and cardiovascular illness, soluble fiber like gums, pectin, and β‐glucan is important for the reduction of blood sugar and uptake of cholesterol and triglycerides (Chuwa et al. 2020). Soluble fiber also affects gut microflora, and it is possible to metabolize it into short‐chain fatty acids that affect human health (Guan et al. 2021). Flaxseed is rich in proteins and peptides that can contribute to human health by virtue of its antifungal, antioxidant, blood pressure‐reducing, and anti‐inflammatory actions and help avoid neurodegenerative disorders. Antidiabetic activity is exhibited by flaxseed hydrolysates. Cyclolinopeptides, a type of peptide in flaxseed, exhibit immunosuppressive, antimalarial, antioxidant, antithrombotic, and antifungal activities (Lorenc et al. 2022).

The most common bioactive compounds that are found in flaxseed are the alpha‐linolenic acid (ALA), lignans, and fiber. The four most common forms of flaxseed that a human being can consume are whole flaxseed, ground flaxseed, flaxseed oil, and partially defatted flaxseed meal. Among the effects of flaxseed supplementation are inflammation, atherogenesis, lipid profile, platelet aggregation, arrhythmias, hypertension, oxidative stress, estrogen activity, and glycemic status (Parikh et al. 2018). Lignans can attach to the body's estrogen receptors and interfere with the estrogenic activity since they are phytoestrogens. Enterolactone and enterodiol were studied to demonstrate anti‐inflammatory, anti‐cancer, and antioxidant effects (Ijaz 2023). The influence and future benefits of lignans and their metabolites can likely be different because of the genes, diet, and the composition of microbiota of an individual. Like any other food item, flaxseed has high amounts of phytoestrogens like ALA in the form of flaxseed flour or flaxseed oil. The Ω‐3 fats attach to the body's estrogen receptors and suppress aromatase action; they possess anti‐inflammatory properties (Imran et al. 2024).

#### 2.1.1 Anti‐Inflammatory Potential of Flaxseed and Flaxseed Oil in Metabolic Disorders

Inflammation is a cellular response, whether to endogenous or exogenous causes, and is thereby a physiologic process aimed at the removal of the causative agent of cell damage and its consequences. It forms the basic etiological factor in several chronic diseases, such as osteoarthritis, insulin resistance, cardiovascular diseases, and many types of malignancies (Chera et al. 2022). The most prominent lignans of flax include those of lariciresinol diglucoside (LDG), matairesinol (MAT), secoisolariciresinol (SECO), and secoisolariciresinol diglucoside (SDG). The coisolariciresinol (SECO) and its diglucoside (SDG) have antiviral, antibacterial, and antifungal properties as well as antioxidant and anti‐inflammatory effects (Khan et al. 2020). Because of the anti‐inflammatory properties of such foodstuffs, the high amounts of omega‐3 fatty acids are considered to be a beneficial approach in this respect. Some of the ways suggested include reducing inflammation in the adipose tissue, promoting adipogenesis, and managing adipokines such as adiponectin and leptin. Flaxseed oil is high in healthy fatty acids and contains high levels of alpha‐linolenic acid (ALA), which may also reduce inflammation substantially. It is possible to mention that this source of ALA has potential health value in terms of being anti‐inflammatory, anti‐atherosclerotic, anti‐chemotactic, and antioxidant. These studies have provided adequate support for the effectiveness of flaxseed eating on alleviating inflammation; however, there are no substantial findings regarding its benefits to overweight prediabetes patients. Thus, the present research aims to determine the impact of flaxseed oil on the level of inflammatory markers and body mass index in pre‐diabetes individuals with excess weight.

#### 2.1.2 Flaxseed Consumption and Prostate Health Outcomes

Pre‐operative studies in prostate cancer patients have shown that linseed supplementation exhibits significant anti‐proliferative effects on tumor cells (Demark‐Wahnefried et al. 2008). One of the possible ways the drug acts may be the down‐regulation of Ki‐67 expression. Targeted genes included powerful oncogenes, suppressors of inhibitory cytokine receptors, key development signaling mediators, proliferative and anti‐apoptotic proteins, and steroid receptors overexpressed in cancer and down‐regulated by flaxseed (Stepień et al. 2025).

In their meta‐analysis approach, the authors concluded the positive role of flaxseed and their derivatives in the indicators of clinical trials for inflammation on the basis of 32 randomized controlled trials (Rahimlou et al. 2019). This is crucial in the advancement of prostate cancer, among other inflammations. Meta‐analysis results showed that HS‐CRP and tumor necrosis factor alpha (TNF‐alpha), inflammatory markers, were reduced significantly following the consumption of flaxseeds. Nonetheless, the intake of flaxseed did not show any substantial improvements in the concentrations of interleukin‐6 (IL‐6) or conventional C‐reactive protein (CRP), yet subgroup investigations showed some (but not much) relief in the levels of IL‐6 concentration. These compounds, the presence of lignans and omega‐3 fatty acids, were attributed to the anti‐inflammatory effects, as inflammatory mechanisms play major roles in leading to cancer and chronic illnesses. Such effects are more evident in the case of people with high BMIs or high baseline inflammation, so it is believed that flaxseed has to be included in the control of prostate health (Rahimlou et al. 2019).

Flaxseed is rich in proteins, fiber, alpha‐linolenic acid (ALA; C18:3, ω‐3), and lignans phytoestrogens that have shown potential in preventing the onset and progression of prostate cancer. In an in vitro study, flaxseed extract upregulated apoptotic‐ and cell death‐related genes such as TNF, CASP8, and Bcl‐2, which resulted in accelerated cell death (mainly necrosis, to a lesser extent apoptosis) in the human prostate cancer PC‐3 cell line, thereby reducing cancer cell viability and impeding tumor growth pathways via modulation of apoptotic and cell‐cycle control systems (e.g., Cyclin D1 and CDK4; Hakimi et al. 2024).

#### 2.1.3 Hypoglycaemic Effects of Flaxseed and Its Bioactive Constituents

Flaxseed has hypoglycaemic effects, lacking serious side effects. Flaxseed contains dietary fiber in a ratio of almost 30:70 of soluble to insoluble concentration, 32.3% fats, 14.1% proteins, 9.8% carbohydrates, and phenolic compounds. The functional constituent of flaxseed, such as alpha‐linolenic acid, soluble and insoluble fiber, phenolic acids, lignans, flavonoids, and tocopherols, in people with metabolic syndrome, provides several medical advantages that are attributed to the improvement of the disease. In patients with type 2 diabetes, a meal containing carbohydrates but supplemented with 15 g of ground raw golden flaxseed seeds reduced the 2‐h postprandial glycemia by 24% and the maximum glucose increase by 17% preliminarily compared to the same traditional meal, according to a randomized crossover clinical trial in men with type 2 diabetes. This was done without a change in meal palatability. Because of the fiber content of flaxseed that slows the emptying of the stomach, raises the viscosity of the digesta, and delays the carbohydrate digestive process, the absorption of glucose by the intestinal brush border was hindered, constituting the primary mechanism of reduced glycemic excursion. Insoluble fiber has been suggested to operate through the following mechanisms: interfering with the digestion of proteins, enhancing excretion of branched‐chain amino acids in the stool, suppressing the formation of glucose in the liver, increasing bowel turnover, inhibition of gastrointestinal carbohydrate‐metabolizing enzymes (alpha‐glucosidase, alpha‐amylase), and modification of the microbiota in the gut (Moreira et al. 2022).

#### 2.1.4 Cardioprotective Effects of Flaxseed on Cardiometabolic Health

Flaxseed doses taken are an effective addition to dietary interventions for healthy metabolism. They are associated with significant improvements in cardiometabolic risk factors. The consumption of flaxseed regularly has been found to have a favorable impact on body mass index (BMI) and weight. This maintains a healthy weight and alleviates the possibility of obesity. The flaxseed helps in the reduction of lipid levels, triglycerides, LDL, and total cholesterol. Nevertheless, the quantity and preparation of flaxseed consumed may affect these impacts; for instance, various lipid parameters might be influenced differently by flaxseed oil and ground flaxseed. Flaxseed has shown high levels of anti‐inflammatory and antioxidant activity. This is evidenced by changes in the key biomarkers such as malondialdehyde, tumor necrosis factor‐alpha, or TNF‐alpha, high‐sensitivity C‐reactive protein, and interleukin‐6. These characteristics depict how flaxseed may help address two of the most important factors of cardiometabolic diseases: oxidative stress and chronic low‐grade inflammation. Linseed is especially helpful for persons at risk of or with type 2 diabetes, as it also improves the management of blood glucose. Its lignans and ALA improve the metabolism, and its high fiber content prevents the uptake of glucose. One of the most observable effects of flaxseed is its ability to lower blood pressure. Both SBP and DBP have consistently been demonstrated to decline by a substantial amount in the studies (Kunutsor et al. 2025).

#### 2.1.5 Anti‐Cancer Potential of Flaxseed and Its Bioactive Lignans

Numerous tumors are already being treated with flaxseed in animal experiments. Breast cancer is probably the most studied cancer in terms of the influence of dietary flaxseed. Consumption of dietary flaxseed has been shown to greatly lower the incidence of breast cancer both in humans and in animal models. Flaxseed reduced tumor growth among cancer patients with breast cancer, as presented in a detailed review of cancer patients involving 10 human studies on patients with breast cancer in general (Calado et al. 2018; Mason and Thompson 2014). Additionally, they discovered evidence linking flax to protection against primary breast cancer and a lower risk of death for breast cancer patients (Flower et al. 2014). Because of their similar chemical structure to mammalian estrogens, flaxseed lignans, which are nonsteroidal phytoestrogens, give animals estrogen‐like effects. Intestinal bacteria break down flax lignans so they can be accessible in plasma. Food‐derived lignans are mostly glucosides that are deglycosylated by gut microbiota β‐glucosidases before intestinal absorption. This hydrolysis releases secoisolariciresinol (SECO) from secoisolariciresinol diglucoside (SDG). After SECO is deglycosylated, the microbiota demethylates it to create dihydroxyenterodiol, which then dehydroxylates it to create enterodiol.

The study aimed to enhance therapy responsiveness and minimize side effects, as well as assess quality of life and overall survival. As observed by the researchers, when the effect of flaxseed lignans on the two breast cancer cell lines (SKBR3 and MDA‐MB‐231) was determined, they potentiated the lethal mechanism of the chemotherapeutic drugs. Also, in a three‐chemical combination trial, enterolactone and metformin at reduced concentrations of chemotherapeutic agents were found to be more effective in inhibiting cancer cell viability than the drugs when used separately. This shows that it would be a worthy attempt to increase the efficacy of chemotherapy on breast cancer (Di et al. 2018).

The mechanisms through which milled flaxseed acquires beneficial anti‐oncotic activity have been studied in human trials, animal models, and cell culture. DG lowers overall death by 40%–53% and death caused by breast cancer by 33%–70%. Antitumor activity is unlikely to be due to DG, however. DG is not taken into the blood and remains only in the intestines. Enterolignans enterolactone and enterodiol are made when it is broken down in the gastrointestinal tract (Edel et al. 2016; Flower et al. 2014). They possess antioxidative and estrogenic activities. Enterolactone and enterodiol are able to alter cell growth by binding to estrogen receptors. These SDG metabolites are likely to be what confer SDG its antitumor activity eventually (Mali et al. 2019). Some of the cellular pathways in the development of cancer that flaxseed and its bioactive ingredients influence include estrogen metabolism, cellular proliferation arrest, angiogenesis, metastasis, inflammation, and the initiation of apoptosis of the tumor (Chen et al. 2009).

#### 2.1.6 Impact of Flaxseed Consumption on Mental Health, Fatigue, and Neurodevelopment

Research has shown that the intake of flaxseed can greatly decrease mental fatigue and improve the mental activity of overweight children and adolescents, as well as have a lesser effect on physical fatigue but may also have a minimal effect on growth, as in a lowered growth in height during peak periods of growth (Gholami and Akhlaghi 2020). The psychological state of depressed women and brain‐derived neurotrophic factor (BDNF) may be connected to flaxseed oil consumption. Two groups were randomly selected from among the research participants. During 10 weeks, the first group was receiving a 1000 mg flaxseed oil capsule twice a day and the second group was receiving a placebo. Before and after the intervention, anthropometric measures of the body and serum BDNF levels were determined. Participants who were participating in research on flaxseed oil supplements saw their depression signs cool down or disappear altogether (Poorbaferani et al. 2020).

Extreme exhaustion brought on by mental and physical effort is known as fatigue. Fatigue is a prevalent issue in many regions of the world. Reports on the incidence of weariness over the past few years have ranged from 33% of Singaporean taxi drivers (Reference Lim and Chia) to 58% of US industrial workers and 81% of Japanese internal medical students. Numerous clinical studies have proven that n‐3 fatty acids are effective for tiredness. Flaxseed is among the highest plants that contain 23 g per 100 g of alpha‐linolenic acid of seeds. Flaxseed may offer benefits beyond its anti‐inflammatory properties, such as reducing hunger. Since both sadness and exhaustion are linked to elevated inflammation, they are connected. Patients with autoimmune diseases frequently experience tiredness and depression; treating inflammation may help them feel less depressed and fatigued (Gholami and Akhlaghi 2020).

In accidents of neuro diseases, flaxseed can also improve some aspects of brain activity through the diet. Under the effect of flaxseed consumption during the period of pregnancy, the loss of brain mass was inhibited, motor hyperactivity and spatial memory were improved, thus preventing the symptoms of depression in children and demonstrating neuroprotection in newborns with hypoxic–ischemic encephalopathy in experimental animals (Mucci et al. 2015). On the basis of such findings, ALA found in flaxseed may be equally important to brain functions. The brains of newborn pups weighed more, and there was a higher concentration of ALA and DHA when their mothers were receiving flaxseed during pregnancy. The usefulness of milled flaxseed, especially in maintaining early postnatal brain development, was apparently shown by the fact that the brains of pups fed milled flaxseed or flaxseed oil shortly after birth had an increased mass (Fernandes et al. 2011).

#### 2.1.7 Antihypertensive Potential and Blood Pressure‐Lowering Mechanisms of Flaxseed

The SBP of 140 mmHg or a DBP of 90 mmHg is identified as hypertension. Hypertension is a prominent precipitator of cardiovascular and cerebrovascular disease. The SBP of greater than 115 mmHg has been proposed as the leading cause of death in the contemporary world. To be eaten on its own or incorporated into various food dishes, the seed makes a pleasant, nutty taste. The antiatherogenic potential, the anti‐inflammatory effects, improvements in vascular contractile physiology, and the strong antiarrhythmic effect of ischemia challenge of flaxseed have seen an immensely high potential to control cardiovascular diseases (CVDs) in animal studies. The composition of flaxseed could make it achieve these cardiovascular effects. The effectiveness of flaxseed has been poorly studied in a patient population, although such findings provide additional insight that is strong in supporting its use in CVD (Rodriguez‐Leyva et al. 2013).

Only when combined with a high‐fat diet has dietary supplementation with SDG resulted in a notable reduction in SBP. Although intravenous SDG might be a helpful pharmaceutical treatment, the anti‐hypertensive effects of eating flaxseed are probably going to happen through the metabolites. The gut microbiota transforms SDG into its aglycone, SECO, rather than circulating it in the conjugated form (Caligiuri et al. 2014). DG increases guanylate cyclase activity and decreases angiotensin converting enzyme (ACE); FPH attenuates renin and ACE activity, and alpha‐linolenic acid in flax oil inhibits soluble epoxide, thus conflagrating blood pressure. The impacts of FLC, flaxseed, and oil flax on blood pressure vary in the context of being negligible to significant. The incompetence of flaxseed and its constituents to reduce blood pressure could be attributed to small amounts of dosage, prolonged intervals of intake, short periods of consumption, and the case of the patients. They will reduce blood pressure, especially in persons with high blood pressure and metabolic syndrome, but are not effective when used in healthy individuals (Prasad 2019).

Flaxseed and its components can reduce blood pressure by a plethora of ways. It was indicated that α‐linolenic acid inhibition in soluble epoxide hydrolase activity minimizes oxylipins, hence medicating the flaxseed antihypertensive properties. The vasoconstriction side effect of some oxylipins has been documented. Thus, a reduction in oxylipin levels would reduce blood pressure. SDG produces a hypotensive effect which is mediated by ACE inhibition according to previous studies. An additional constituent of flaxseed is protein. It has been established that inhibition of renin and ACE action is the mechanism through which flaxseed protein hydrolysate produces hypotension. The results suggest that hypotensive actions of flaxseed could be carried out through reductions in oxylipin levels, the activation of guanylate cyclase, and ACE and renin inhibition (Prasad 2019).

**FIGURE 3:**
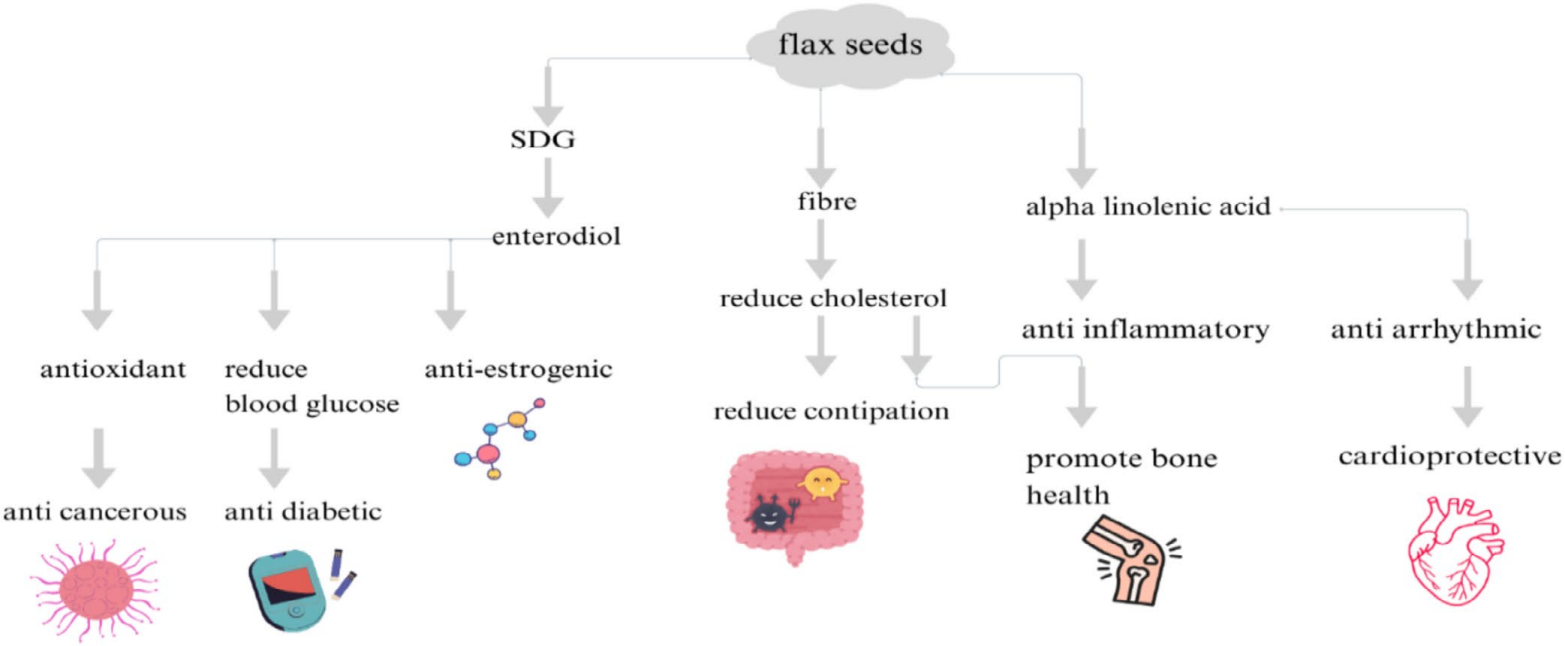
The health benefits of bioactive compounds in flaxseeds.

The health impacts of bioactive compounds in flaxseeds are shown in Figure 3, as SDG, fiber and alpha‐linolenic acid are beneficial against cancer, inflammation, oxidative stress, reduce constipation, are cardioprotective, and promote bone health.

### 2.2 Chia Seeds

Chia seed, *Salvia hispanica L*., is a South and Central American oilseed pseudo‐cereal. Chia seeds are mainly produced in Guatemala, Bolivia, Peru, Ecuador, and Mexico. They are found in foods and medicines, as well as in the manufacture of paint. Chia seeds recently found popularity in countries like Australia, Canada, the USA, and Japan. They are oval and flat in shape and measure 2.0–2.5 mm in length, 1–2 mm in width, and 0.8–1.0 mm in thickness. They contain a wide range of vitamins, minerals, and vital nutrients like phytochemicals (Ashura et al. 2021). The use of chia seeds prevents the onset of diseases like diabetes, obesity, cardiovascular diseases and other cancerous diseases because of the chemical composition, including dietary fibers, proteins, essential fatty acids, vitamins, minerals, carotenoids, anti‐inflammatory, and antioxidant compounds (Grancieri et al. 2019).

Its oil is also high in polyunsaturated fatty acids and other micronutrients. PUFAs comprise approximately 25%–35% of the oil composition. Its high fiber composition has been noted to reduce cholesterol, alter insulin levels, and reduce the glycemic index and have a beneficial effect for type 2 diabetes (Rendón‐Villalobos et al. 2012). The environment and the processing of extricating those compounds may have an effect on its nutrient elements that have therapeutic effects used as nutraceuticals. Chia seeds contain 10 essential amino acids in large proportions including phenylalanine, arginine, and leucine, among other amino acids. It does not contain gluten, and this characteristic makes it appropriate in cases of patients who are celiac. It is composed of nutritious minerals, including magnesium, zinc, iron, calcium, phosphorus, and potassium. These nutrients are healthier than wheat, rice, and oats (Muñoz et al. 2013). It includes bioactive compounds, such as catechin, chlorogenic acids, quercetin, etc., and essential fatty acids that include polyunsaturated fatty acids, including those that contain omega‐3 fatty acids to a significant level (Ghafoor et al. 2018). Polyphenolic chemicals, which are high in chia seeds, make up about 8.8% of the total seed components (Knez Hrnčič et al. 2019).

#### 2.2.1 Anti‐Inflammatory and Immunomodulatory Effects of Chia Seed Bioactive Peptides

Chia seeds are also a good source of bioactive peptides since they have all the requisite amino acids and proteins. These peptides can exert an influence on the body's physiological processes, positively affecting them depending on the composition and the structure of the amino acids, contributing to immunity activation. To exert their immunostimulatory effect, bioactive peptides are able to control specific and non‐specific immunity (Villanueva‐Lazo et al. 2022). The mechanism of action is to actively increase the number of cells that produce IgA‐type antibodies, the synthesis of immunoglobulins, and the cell's ability to phagocytose. Experiments have revealed that the principle adapted by chia seed peptides is relevant to oxidative tussles in the body, adipogenesis, and the inflammatory processes. Because of inhibition of adipogenesis by blocking the expression of proteins as a result of protein digestion, fractions of protein may inhibit adipogenesis by suppressing the expression of the peroxisome proliferator‐activated receptor‐γ (PPAR‐γ) in the mature adipocyte. They also lowered lipoprotein lipase, fatty acid synthase, and sterol regulatory element binding protein 1 activity as well (Grancieri et al. 2021).

In rats modeling, monocytes can differentiate into two main phenotypes: the pro‐inflammatory profile of the M1 cells and encouragement of the oxidative stress, and the anti‐inflammatory and antioxidant capability of the M2 cells, which can be characterized by the presence of the surface markers CD14 and CD16, and which play a compulsory role in the mediation of immune responses and the pathogenesis of chronic diseases (Villanueva‐Lazo et al. 2022). Many evidences showed PAMP pathway recognition involving LPS molecules and pathogen recognition receptors, for example, toll‐like receptors (TLRs), are macromolecular elements crucial to the innate immune system and can activate macrophages through nuclear factor‐kappa B (NF‐kB) activation (Liu et al. 2017). Therefore, interferon gamma (IFN) is one of the strongest activators of macrophages (M1 macrophages) and the source of pro‐inflammatory and oxidant content (Wang et al. 2018). The synthesis of the inflammatory cytokines generates the inflammatory state that coexisted with the catabasis of the adaptive immunity and that of non‐classical macrophage activation (M2 macrophages), with IL‐4 being one of the greatest triggers of the latter (M2 macrophages; Yu et al. 2019).

#### 2.2.2 Role of Chia Seed Omega‐3 Fatty Acids in Brain Health and Neuroprotection

Chia seeds are rich in vital omega‐3 and omega‐6 polyunsaturated fatty acids, including omega‐3 PUFA alpha‐linolenic acid (ALA). Both EPA and DHA possess neuro‐protective effects, including reducing neuroinflammation, and are critical to numerous intra‐brain processes, including signal transduction. Alzheimer's disease patients have a shift in PUFA metabolism (Schreyer et al. 2020). The findings showed that in an increase in the amount of ALA ingested, higher concentrations of the omega‐3 fatty acids particularly DHA occur in the brain and other tissues of the offspring. The upregulation of genes and proteins involved in DHA transportation and the greater DHA phospholipid content of the brains of ALA offsprings may be the cause of the improved memory performance of the ALA offsprings at 19 weeks of age. Moreover, unlike other nutrients, ALA affects the development of the brain positively in old people besides contributing positively to the developing brain of young population. AA and DHA are manufactured by the conversion of ALA and LA, respectively. Aging has been found to influence fatty acid desaturase activity with a greater preference of aging individuals to ALA compared to LA as the substrate affinity increased. Thus, ALA supplementation elevates DHA, not LA, elevating AA, which can protect the brain by sparing brain neurons and, therefore, protecting against neurological disorders such as AD (Leikin‐Frenkel et al. 2022; Zhu et al. 2024).

#### 2.2.3 Cardioprotective Effects of Chia Seeds in Cardiovascular Disease Prevention

Acute and chronic heart disease, arrhythmia, heart failure, stroke, and arterial hypertension are some of the CVDs, constituting a large percentage of non‐communicable diseases (Münzel et al. 2021). One of the main causes of heart disease is diet. Diet‐related risks include obesity, hypertension, ineffective management of hyperglycemia, and the presence of animal fats in cooked meals and foods in large quantities. The organic food, soluble fiber, and plant lipid in the diet could result in reducing the risk of coronary heart disease (Bechthold et al. 2019).

All cells have cholesterol, a waxy substance with many helpful characteristics, such as the capacity to modify the shape of human cells. It is disseminated through the lipoprotein that is bound to blood (Cook 2015). Following precipitation of apo‐B‐containing lipoproteins, HDL‐C, commonly expressed as mg/dL, is measurable directly from serum by enzymatic assay using the cholesteryl esterase and cholesterol oxidase reaction (Cho 2022). Increased concentrations of circulating HDL‐C have been stated to be inversely proportional to an individual developing CVD (Grao‐Cruces et al. 2021). Saturated fats and vegetable oils with excessive polyunsaturated fatty acids reduce the TC: HDL‐C ratio. It also reduces the incidence of coronary heart disease by enriching omega‐3 unsaturated fatty acids in vegetable oils (Flock and Kris‐Etherton 2012).

The seeds are highly significant in human nutrition. Significantly, a larger portion of the available evidence relies on the positive effects of the increase in usage of plant seeds and their byproducts on several health parameters. A matrix with a complex outer layer and a germ that is full of bioactive phytochemicals, vitamins, and minerals, which comprises the seed, can defend the plant DNA against oxidative stress and can help maintain the existence of the species. Omega‐3 in chia seeds lowers cholesterol, fiber, minerals, and vitamins are hypotensive in nature. Antioxidants and vitamins reduce the levels of oxidation, thus reducing the occurrence of CVD (Khalid et al. 2022).

#### 2.2.4 Anti‐Cancer Potential of Chia Seed Phenolic Compounds

**FIGURE 4:**
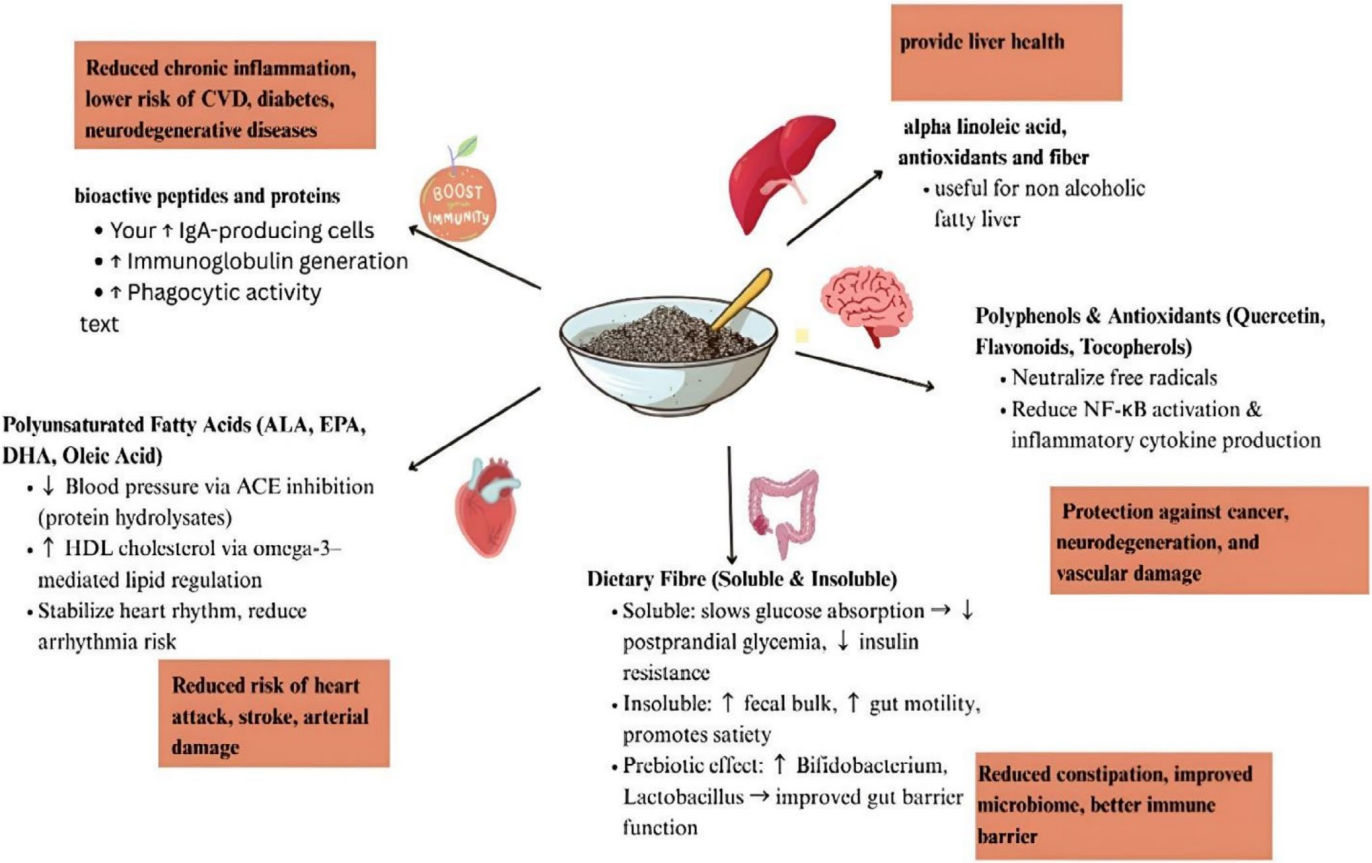
The nutraceutical benefits of chia seeds.

Phenolic compounds appeared as the combination of hydroxybenzoic and hydroxycinnamic acids. Examples of hydroxybenzoic acids include Protocatechuic, p‐hydroxybenzoic, Vanillic, Gallic, and syringe acids. In the interim period, caffeic, p‐coumaric, and ferulic hydroxycinnamic acids were demonstrated to contain aromatic components. Since phenolic compounds have the ability to donate hydrogen or electrons in addition to delocalizing the unpaired electron in the reactive structure, they can also scavenge free radicals and chelate metal ions as part of their antioxidant action. To establish the anti‐cancerous activity of Chia seeds ( *Salvia hispanica* ), this study used breast cancer MCF‐7 cells and evaluated the plant to inhibit the growth of cancer cells. The MCF‐7 human breast epithelial cells demonstrated a significant, concentration‐dependent cytotoxic effect on exposure to chia seeds ( *Salvia hispanica* ) at concentrations of 0, 25, 50, 100, and 200 g/mL following 24 h of exposure. It exhibits the possible anti‐cancer treatment of chia seed ( *Salvia hispanica* ) extract. Chia seeds are novel foods utilized in many countries and do not have adverse or allergenic effects on the health of the population. The present study relates to other studies that increased the anti‐cancer and antioxidant effect of chia seeds, and hence, this initial work supplements and ensures the immense potential of research in this field to deliver an effective drug against breast tumors in the interest of humanity (Mutar and Alsadooni 2025), as illustrated in Figure 4.

### 2.3 Sesame Seeds

Sesame (*Sesamum indicum L*.) was found in Pakistan. India, Myanmar, Sudan, China, and Tanzania are the largest producers of sesame seeds, and in the last few years, Tanzania has become the largest grower of sesame seeds. Around 5.899 million tons was harvested in 2017, and out of that, Tanzania produced 806,000 tons, and China produced 733,000 tons. There is white sesame, black sesame, and yellow sesame on the basis of germplasm color (FAO 2018). Oil content has a positive relation with germplasm color, as the color of white sesame contains the maximum amount of oil, and as the color darkens or becomes deeper, the oil content decreases (Wei et al. 2022). It has protein, saturated fats, and calcium, iron, copper, vitamin E, vitamin B1, and magnesium. It has antioxidants that render it stable for a long duration since it can withstand oxidative rancidity (Agidew et al. 2021). Mn, Mg, Cu, Fe, and Zn are metals that the human body requires to perform its healthy functioning is explained by their participation in the metabolic reactions. It is also a source of sesamin and sesamolin, which are lignans, potentially dietary fibers and offer protection against hypertension, preventative against vascular degeneration, and help to lower cholesterol levels (Beshaw et al. 2022).

Two phenylpropanol molecules combine to generate lignans, which are metabolites. In sesame, furofuran core (3,7‐dioxabicyclo [3.3.0] octane) is prepared by fusion of oxopropane side chains of cinnamoyl alcohol to synthesize lignans. Furofuran lignans are the name given to these metabolites. Sesamin may possibly be turned into a medicinal treatment because of its capacity to inhibit tumor development. Creams and body oils include lignans and sesame oil (Andargie et al. 2021). Nutritionally significant natural antioxidant lignans, including sesamin, sesamolin, sesaminol, and sesamol. The two main phenolic chemicals that are unique to sesame plants are sesamin and sesamolin. Sesame seeds can contain somewhere between 1.4% and 1.4% of these two lignans overall. The little amounts of lignans that are present, however, are mostly the result of chemical changes that occur during the processing of sesame seeds and oil. For instance, sesame seeds mostly contain glycosylated forms of sesaminol (Sintim 2024).

#### 2.3.1 Anti‐Inflammatory

Atherosclerosis begins with the deposition of cholesterol crystals in the artery's inner layer (intima) and underlying smooth muscle. These deposits form plaques that grow, surrounded by fibrous tissue, which narrows blood flow. The process is driven by chronic inflammation and oxidative stress caused by factors such as nitric oxide synthase, myeloperoxidase, xanthine oxidase, lipoxygenase, cyclooxygenase, and NADPH activation. This oxidative damage, along with elevated blood sugar and lipids, leads to plaque buildup and arterial narrowing. Inflammatory cells accumulate, smooth muscle cells proliferate, and fibrous tissue forms, further contributing to vessel blockage (Hadipour et al. 2023). Despite not being inherently atherogenic, low‐density lipoprotein (LDL) cholesterol could become atherogenic in an oxidized state. The main finding is the pattern of the fixation index that is close to unity, since it is said that the fixation index is near unity when brother–sister mating patterns are nearly uniform (Linton et al. 2019). In addition, an increase in the antioxidant enzymes glutathione peroxidase, superoxide dismutase, and catalase was noted; the patient also has reduced antioxidant status and a decline in E, A, and C vitamins in patients with atherosclerosis (Lubrano and Balzan 2015).

#### 2.3.2 Neuroprotective Effects of Sesame Seed Lignans in Brain Health

Some of the minor nutrients contained in sesame seed oils include proteins, unsaturated and saturated fatty acids, and lignans, such as sesamin, sesamol, sesamolin, and tocopherols. The seeds are also rich in iron, magnesium, copper, calcium, vitamin B1 (thiamine), and vitamin E (tocopherol) as well as phytosterols. Because of its strength in resisting oxidative rancidity even after a long period of exposure to air, sesame seed oil is renowned as very durable (Agidew et al. 2021). Oxidative stress is an excess of free radicals which disrupts homeostasis and balance of antioxidants. The redox process keeps that balance of free radicals' production and removal. Sesamin is known to contain antioxidant properties as well as successfully eliminating free radicals in the body. In human neuroblastoma, sesamin prevented the synthesis of reactive oxygen species (ROS) elicited by H_2_O_2_. To protect the cells against oxidative stress, the enzymatic activity of catalase (CAT) and superoxide dismutase (SOD) was enhanced as well. Sesamin managed to revert the oxidation‐induced decline in SIRT1 and SIRT3 expression. It has been demonstrated that sesamin suppresses the oxidative damage caused by H_2_O_2_ and also has the effect to modulate the SIRT1‐SIRT3‐FOXO3a signaling pathway (Abdulkhaleq 2024).

Sesamin is an anti‐inflammatory and antioxidant neuroprotectant that is found in sesame seeds. It can also suppress the release of cytokines by microglial cells, protect the integrity of the blood–brain barrier, and reduce the amount of neuron injury in animal models of brain injury and neurodegenerative diseases. Sesamin also has anti‐activation of microglial response, oxidative stress, and inflammation and consequently enhances its prospects in preventing stress‐induced cognitive impairments and psychiatric disorders. Although such positive results are found, additional research is needed to support definitively the neuroprotective effect of sesamin (Zhao et al. 2019).

Sesamol, which is one of the key lignan compounds of the sesame seeds, has antioxidative, anti‐aging, anti‐inflammatory, antimutagenic, and chemopreventive effects. It also regulates key signal transduction pathways, such as p53, MAPK, PI3K/AKT, NF‐kB, and caspase‐3, which promote antioxidant countermeasures, antitumor effects, and programmed cell death. Empirical studies establish that sesamol is a potent antioxidant against free radicals, reduces oxidative stress, and changes cellular progression, and thus is a strong choice as a preventative and curative agent of cancer (Pal et al. 2020).

#### 2.3.3 Anti‐Cancer Mechanisms of Sesamin and Sesamolin in Sesame Seeds

Because of its antiproliferative, proapoptotic, antimetastatic, and pro‐autophagocytic action, sesamin, present in sesame seeds, exhibits anti‐cancer properties. It acts as a metabolic regulator endowed with antimutagenic, antihepatotoxic, and anti‐inflammatory effects while inhibiting the pathways towards tumor growth and progression (Karlsen et al. 2016). Sesamolin extracted from sesame seeds causes death in human lymphoid leukemia cells through DNA fragmentation. Sesamin inhibits proliferation at early stages of the G1 cell cycle (Jaffar et al. 2025). Arresting the cell cycle at the G 1/S phases forms an important means of inhibiting cancer growth. The MAPK/ERK pathway is also disturbed, causing apoptosis stimulation. The process changes histone methylation and acetylation and alters the way a gene is expressed (Wang, Yen, et al. 2020). Sesamin reduces angiogenesis as well as tumor microvessel density through the inhibition of vascular endothelial growth factor (VEGF) formation (Meng et al. 2021). Sesamin helps to suppress the expression of the estrogen receptor α (ERα) and androgen receptor (AR). It also suppresses the matrix metalloproteinases (MMPs). Sesamin enhances antibody‐dependent cellular cytotoxicity and autophagy (Jaffar et al. 2025).

**FIGURE 5:**
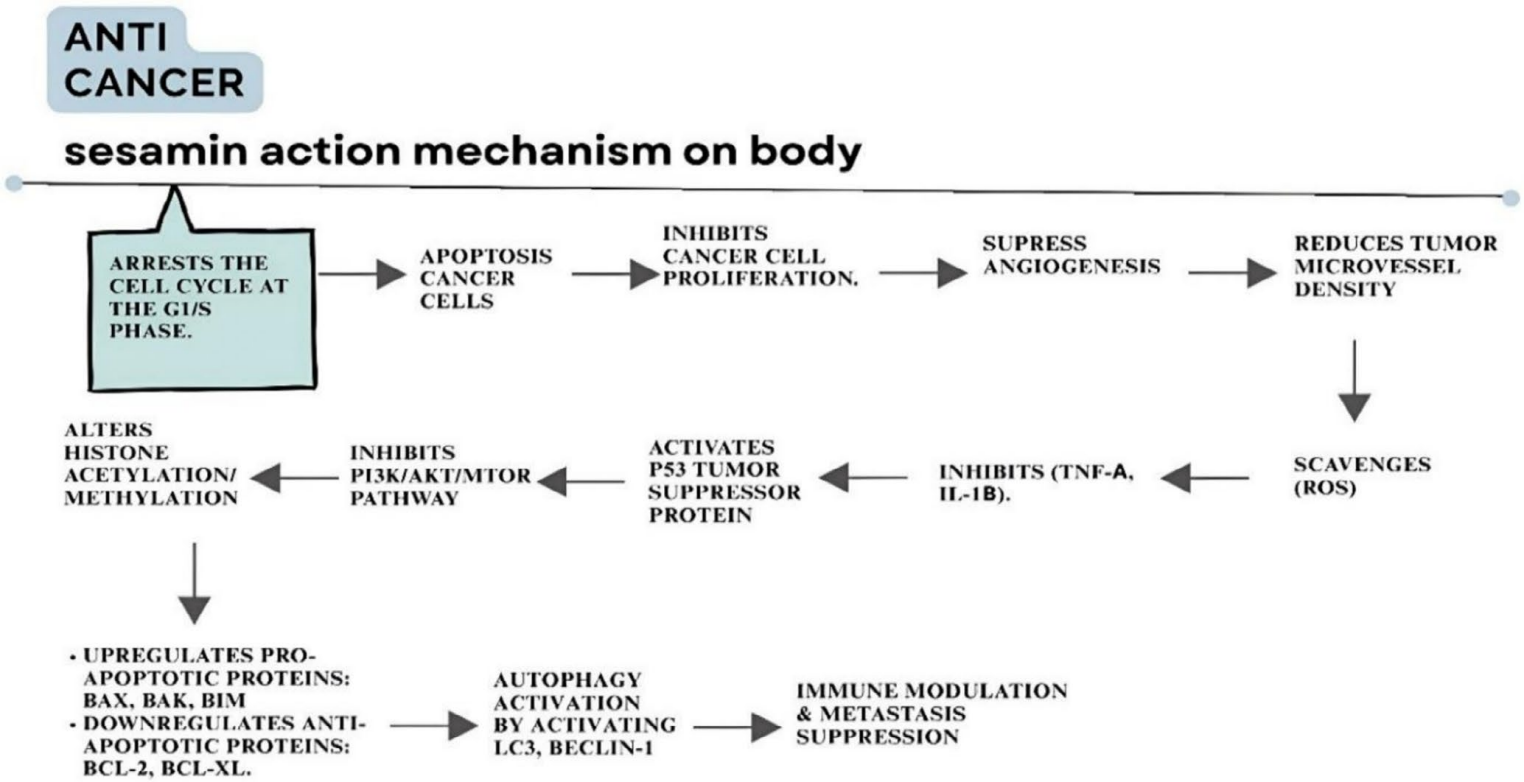
The mode of action of Sesamin against cancer.

Sesame seed extract is rich in a bioactive lignan, sesamin, that is protective against cancer, and its mechanism of action is illustrated in Figure 5. The arrest of the cancer cell cycle at the G1/S phase initiates the action. Cancer cell apoptosis halts the growth and inhibits angiogenesis. This decreases micro‐vessel density of tumor and prevents TNF‐alpha, IL‐beta one. Lowers anti‐apoptotic proteins and upgrades pro‐apoptotic proteins by activating a tumor suppressor protein, P53, which inhibits the PI3K/Akt/mTOR pathway and alters histone acetylation/methylation. Proteins LC3 and Beclin 1 activate autophagy and prevent metastasis.

#### 2.3.4 Cardioprotective Effects of Sesamin in Lipid and Cholesterol Regulation

Sesamin has strong lipid‐lowering activity. Sesamin's action on critical stages of cholesterol and fatty acid metabolism is the central reason why it lowers lipid levels. It achieves this by decreasing levels of TG, VLDL, and LDL that have been known to lead to the development of atherosclerosis. Sesamin also increases levels of HDL, which are said to offer protection against atherosclerosis (Meng et al. 2021). Sesamin was reported to enhance lipid control and cholesterol reduction through the reduction of mRNA levels in genes responsible for cholesterol uptake. It also elevated the levels of CYP7A1 mRNA and reduced HMG‐CoA reductase and LXRα mRNA, indicating its functionality in cholesterol absorption. Sesamin influences various facets of cholesterol metabolism. It reduces HMGC and subsequently depresses CYP7A1 expression and activity and suppresses the formation of cholesterol (Abdulkhaleq 2024).

#### 2.3.5 Hypoglycaemic and Antidiabetic Effects of Sesame Seeds

Because they have high dietary fiber and act as enzyme inhibitors that function by breaking down carbohydrates, sesame seeds possess antidiabetic activity. They also aid in blood glucose control and can be beneficial to people with diabetic nephropathy (Kapoor et al. 2015). Sesame seeds are high sources of bioactive lignans, which have been found to alleviate the management of diabetes through their ability to increase insulin secretion, increase insulin sensitivity through the activation of IRS‐1 and PI3K pathway, and reduce carbohydrate digestion by inhibiting 8‐glucosidase. Also, they have anti‐inflammatory and antioxidant effects that help preserve pancreatic 6 ‐cells, regulate gut microbiota, and enhance glucose homeostasis, partly by stimulating the secretion of GLP‐1 and the metabolism of lipids. Glibenclamide used with sesame seeds has also been reported to have a considerable effect in lowering fasting blood glucose, lowering HbA1c levels, and improving lipid profiles, which means that it can be considered an adjunct therapy with glibenclamide in treating diabetes (Jaffar et al. 2025).

#### 2.3.6 Antihypertensive Effects of Sesamin in Cardiovascular Health

Sesamin has been shown in numerous studies to have potent lipid‐lowering properties. Sesamin's effect on important phases of fatty acid and cholesterol metabolism is the main cause of its ability to reduce lipid levels. It does this by lowering TG, VLDL, and LDL levels, all of which are known to contribute to the formation of atherosclerosis. Sesamin also raises HDL levels, which are believed to protect against atherosclerosis. It may reduce stocks of some sterol transporters and reduce the absorption of cholesterol and enhance neutral steroid production as stool. Sesamin is also helpful in the maintenance of balanced cholesterol levels because it elevated the expression of RCT sterol transporters and triggered the cholesterol regulatory networks of PPARgamma 1, LXRalpha, and MAPK (Abdulkhaleq 2024). The study conducted by (Miyawaki et al. 2009) shows that there has been a potential for sesamin blood pressure lowering in people. On the basis of our studies, 60 mg of sesamin dosage in 4 weeks resulted in the lowering of blood pressure by 3.5 mmHg (systolic blood pressure) and 1.9 mmHg (diastolic blood pressure). This is the first study that addresses the effect of sesamin on blood pressure as of our knowledge.

### 2.4 Melon Seeds

Melon pulp and rind pigments consist of carotenoids and chlorophylls. Proteins and sugars are present in melons. Melons contain antioxidants such as phenolic compounds, ascorbic acid, and carotenoids. Seeds are a good source of vitamins E and omega‐3 fatty acids (Manchali et al. 2021). Melon (*Cucumis melo L*.) has gained a higher rate of production and has an excellent economic value, which in recent times means a loss of a portion of the fruit. Throwing away the edible portions, that is, peel and seeds, is normally practiced at the time of consumption and processing. *Cucumis melo L*. is a fresh fruit that is exported and consumed worldwide, with peel and seeds being thrown away. Melon seeds contain 4.5% moisture, 25.0% crude protein, 2.4% ash, 25.0% crude fat, 23.3% crude fiber, and 19.8% carbohydrates (Franklin 2021). The nutritional content of melon seeds includes 26.26%–39.96% protein, 26.56%–55.0% fat, 1.11–49.54 mg/100 g iron, 0.26–18.58 mg/100 g copper, 0.75–22.09 mg/100 g zinc, and 12.2 μg/100 g selenium (Enujiugha et al. 2023).

*Cucumis melo* has been used in traditional medicine owing to its expected health delivery. Certain civilizations assign the properties of being digestive and diuretic. Underpinnings to the melon. The pulp is normally suggested in order to lessen inflammation and enhance hydration in the case of hot weather. Capsicum melo is purgative in nature. Some of its uses include dysuria, reductions in blood pressure, control over blood functions, dyspeptic issues, flatulence, fever, jaundice, diabetes, obesity, cough, bronchitis, ascites, anemia, constipation, and menorrhagia (Yadav et al. 2025).

#### 2.4.1 Neuroprotective and Cognitive Benefits of Melon Seeds

The seeds of muskmelons contain bioactive ligands like tocopherols, phospholipids, and sterols with anti‐inflammatory, antioxidant, and anti‐Alzheimer properties. The seeds are also high in omega‐6 and omega‐9 fatty acids, which are linked to enhanced hormone levels and even brain activity. Moreover, the melon seeds have some pharmacological effects, such as immune‐modulatory and antidiabetic. Considering its rich nutrition and practical usefulness, melon seeds could become a positive food material and an organic medicine for many conditions, especially those that affect the brain (Tiwari et al. 2025). Some studies have shown that squalene is a marine bioactive substance that has been found to have potential use as a treatment for Alzheimer's disease (AD) when using shark liver oil. Squalene enhances cognition in rats with AD‐like phenotype through the increase in acetylcholine levels and the inhibition of acetylcholinesterase activity in brain areas of memory formation, the hippocampus and the cortex in particular. Moreover, squalene could prevent aggregation of the amyloid plaques and hyperphosphorylated tau protein, which is a characteristic neuropathologic feature of AD and, therefore, potentially mitigate neuronal damage (Praveen and Kuna 2021).

#### 2.4.2 Anti‐Inflammatory and Anti‐Cancer Properties of Melon Seeds

Infectious disorders brought on by bacteria entering the body can induce inflammation, which can also occur in certain tissues or circulate in the circulation. Additionally, tissue damage, cell death, malignancy, ischemia, or tissue degeneration can all cause inflammation (Ezzat et al. 2019). The research was done by (Bouaziz et al. 2020). This was confirmed by the set of results showing that there is a high amount of total phenolic compounds present in the ethanolic extract of melon seeds, which can help the field of functional foods by suggesting a potential new use of the seeds, namely, the prevention of illnesses due to oxidative stress and anti‐inflammatory disorders.

Polyphenols such as flavonoids, phenolic acids, and hydrolyzable tannins have antitumor effects by protecting the genomic integrity against oxidative genotoxicity, neutralizing carcinogenic agents, and promoting the phase I and phase II detoxification cascades. They can slow the progression of tumors by suppressing oxidative stress, inducing apoptosis, and preventing cellular growth, migration, and migration. In vitro tests using extracts of melon residue showed great antiproliferative effect in a range of human carcinoma cell lines with inhibition rate of 20%–85%, especially in cervical, colorectal, and hepatic cancers (Franklin 2021).

#### 2.4.3 Cardioprotective Effects of Melon Seeds

The rise in ROS generation and the fall in antioxidant activity can lead to a number of anomalies. These anomalies include endothelial dysfunction that induces atherosclerosis by reducing the vasodilator bioavailability of nitric oxide (NO) and raising the levels of endothelium‐derived contractility factors (Fernández‐Sánchez et al. 2011). *Cucumis melo* is a source of magnesium, potassium, iron, vitamin C, vitamin A, vitamin B6, calcium, pantothenic acid, zinc, omega‐3, and omega‐6, which are vital nutrients and minerals. Eating sweet melons has been associated with blood pressure control, and this is mainly attributed to potassium. *Cucumis melo* has both soluble fiber and insoluble fiber, and dietary fiber in the fruit is highly helpful in facilitating easy digestion and bowel movement in the body. In effect, *Cucumis melo* assists the body in kicking out harmful LDL‐c and other toxins (Adebayo‐Gege et al. 2022).

#### 2.4.4 Antihypertensive Effects of Melon Seed Bioactive Compounds

The failures expected include atherosclerosis, non‐alcoholic steatohepatitis, and insulin resistance caused by obesity, which are preventable because of a reduction in non‐adipose tissue lipid accumulation by using squalene. To sustain the oral levels, the squalene‐based oil‐in‐water emulsion adjuvants could be used to decrease the cholesterol content in RAW 264.7 macrophages (Cheng et al. 2024). Squalene is a liver X receptor (LXRa and LXRb) agonist that activates transcription of transporters (ABCA1, ABCG1, and apolipoprotein E) to promote cholesterol release into the cell. Examples have been provided through experimental studies in animal models, which have shown that diet squalene administration leads to hepatic accumulation, simultaneous inhibition of hepatic lipid deposition and triglyceride level, without causing hepatic lipogenesis. These results implicate squalene as an inducer of cholesterol homeostasis and propose a possible therapeutic use of squalene in the treatment of hepatic lipid disorders (Hien et al. 2017).

One of the significant studies was done on the antihypertensive potential of white melon seed protein concentrate (WSP) supplemented with sweet orange peel in sodium fluoride‐induced hypertensive rats. The research determined that WSP blocked the activity of angiotensin‐converting enzyme (ACE), which is fundamental to the RAAS framework to convert angiotensin I to the vasoconstrictor angiotensin II. WSP reduced vasoconstriction by inhibiting ACE. Also, WSP increased the availability of NO by reducing oxidative stress. The NO increases in the body result in vasodilation, and this enhances endothelial functioning, which further lowers blood pressure. Besides, WSP inhibited pro‐inflammatory cytokines, including tumor necrosis factor‐alpha (TNF‐α) and interleukin‐1 beta (IL‐1β) that increase vascular inflammation to enhance hypertension (Fasakin et al. 2025).

**FIGURE 6:**
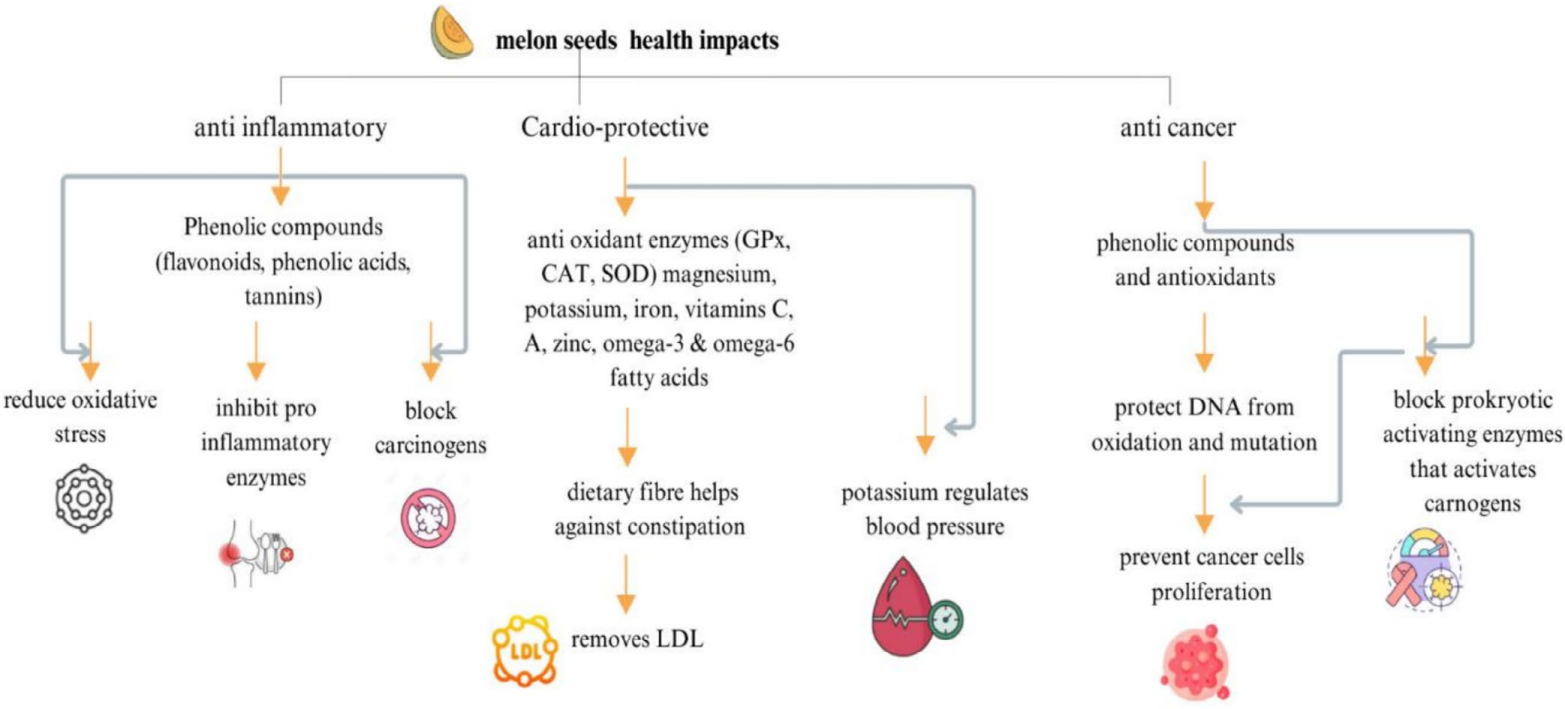
The health benefits of bioactive compounds in flaxseeds.

The health benefits of melon seeds include anti‐carcinogenic, cardioprotective, reducing constipation, and regulating blood pressure because of phenolic compounds, antioxidant enzymes, vitamins, dietary fiber, zinc, magnesium, and potassium, as illustrated in Figure 6.

### 2.5 Pumpkin Seeds

Pumpkin is a vegetable of the *Cucurbitaceae* family and has been used for many years because of its nutrient value (Montesano et al. 2018). The seed of pumpkins, however, is specifically known as pumpkin seeds. The flattened, round seeds contain white hulls and bright green pulp. However, most pumpkin seeds are considered agro‐industrial waste. Their highly rich source of bioactive compounds with a range of nutraceutical properties is fast gaining them attention (Hu et al. 2024).

Pumpkin seeds contain several activities, such as reducing the risk of diabetes, hypertension, tumors, low immunity, bacterial and viral infections, hypercholesterolemia, and analgesic activity (Alasalvar et al. 2021). As linoleic acid is a necessary fatty acid that the body cannot synthesize from other food components, its large amount makes it an important nutritional component of pumpkin seed oil. Vitamin D, various hormones, and cellular membranes are all synthesized with linoleic acid (Sinkovič et al. 2021). Vitamins and substantial levels of minerals, especially iron, potassium, magnesium, and phosphorus, are present in pumpkin seeds. As a result, consuming pumpkin seeds helps individuals who are taking diuretics and lose extra potassium levels. According to the US/Canadian Dietary Reference Intakes, consuming pumpkin seeds can help individuals fulfill the approximate daily protein consumption of 0.9 g of protein/kg/day because they are rich in a significant concentration of proteins (Dowidar et al. 2020).

#### 2.5.1 Anti‐Inflammatory Properties of Pumpkin Seeds

Al‐Okbi et al. (2017) reported that pumpkin seeds possess strong anti‐inflammatory and antioxidant properties because of their bioactive compounds. Pumpkin seeds are sources of bioactive substances, which include unsaturated fatty acids, phytosterols, kaempferol, carotenoids, as well as phenolic compounds and have been shown to possess strong antioxidative and anti‐inflammatory properties. These compounds have the combined effects of dampening inflammation by altering molecular signaling pathways controlling oxidative stress and production of inflammatory mediators. A defining process is a high level of unsaturated fatty acids, especially omega‐3 and omega‐6 fatty acids, which are used as precursors to the anti‐inflammatory eicosanoids. The fatty acids aid in limiting pro‐inflammatory cytokines, which include TNF‐α, IL‐6, and C‐reactive protein (CRP). Also, phytosterols and tocopherols contained in pumpkin seed oil neutralize ROS and thereby antagonize the oxidative stress that secondarily intensifies inflammatory reactions in body tissues. Decreased oxidative stress also decreases the activation of the pro‐inflammatory transcription factor nuclear factor kappa B (NF‐kB), which has down‐regulatory effects on the expression of adhesion molecules and pro‐inflammatory enzyme (Shaban and Sahu 2017).

#### 2.5.2 Anti‐Cancer Properties of Pumpkin Seed Oil and Bioactive Compounds

Triterpenes, phytosterols, phytoestrogens, polyunsaturated fatty acids, tocopherol, carotenoids, and antioxidative phenolic substances are all found in significant concentrations and naturally in PSO. PSO has been shown to have therapeutic promise for prostatic hypertrophy, atherosclerosis, hypertension, and bladder hyperplasia17–19. Alpha‐tocopherol, a potent antioxidant found in abundance in pumpkin seeds, appears to be essential for preventing some illnesses. It has been determined that cellular oxidative stress (COS) is highly contributory to cancer development. Certain mutations in nuclear and non‐nuclear (mitochondrial) genes that encode elements of the electron transport chain can increase the formation of COS, according to the mitochondrial model of cancer (Ghosh et al. 2025). Alpha‐tocopherol, a potent antioxidant found in abundance in pumpkin seeds, appears to be essential for preventing some illnesses. It is proven that cellular oxidative stress (COS) plays an important role in the pathogenesis of cancer. Some nuclear and non‐nucleotide (mitochondrial) gene mutations that code components of the electron transport chain can enhance the generation of COS, as described in the mitochondrial theory of cancer. Epithelial cells of the thyroid gland produce reactive oxygen species (ROS) in quantities which are necessary to synthesize T3 and T4 hormones. Any increment in oxidant factors is associated with the development of thyroid cancer (Ghosh et al. 2025). Development, cellular differentiation, and both physiological and non‐physiological death depend on the process of apoptosis (Li‐Weber 2013).

#### 2.5.3 Cardioprotective Effects of Pumpkin Seeds and Pumpkin Seed Oil

Rich in vitamins, fiber, and carotenes, pumpkin seed oil aids in the control of diabetes and cardiovascular disease. Pumpkin seeds' polysaccharides function as antioxidants, and their high tryptophan level helps to lessen sadness. By lowering oxidative stress and inhibiting plasma lipoprotein oxidation, pumpkin seeds aid in the management of cardiac disorders. Their oil, which is high in phytosterols and unsaturated fatty acids, limits the absorption of cholesterol by raising HDL and decreasing LDL. Because of its antioxidant properties, pumpkin seeds lower the risk of cardiovascular disorders, hypertension, and arteriosclerosis. Their oil significantly raises HDL and decreases blood triglyceride. The phytosterols in pumpkin seeds help lower cholesterol, and regular ingestion helps control blood pressure. applications of extracts from pumpkin and allied species in the pharmaceutical, medicinal, and food industries. The various phytochemicals and possible pharmacological benefits found in the *Cucurbitaceae* family. Their potent antioxidant qualities are attributed to tocopherols, zinc, and selenium (Adnan et al. 2025). The myocardium's poor antioxidant mechanisms and rapid oxygen consumption make it extremely vulnerable to free radicals. The optimal oxygen intake is the outcome of aerobic activities and enhances the volume of blood and plasma, blood pressure, and antioxidative enzyme level. Pumpkin fruit has a very high nutritional value and their content includes antioxidants such as beta carotene (a great source of vitamin A), vitamin E, vitamin C, salts and minerals such as selenium, calcium, manganese, magnesium, potassium, iron, and zinc, and unsaturated fatty acids such as omega‐3 and omega‐6 fatty acid including linoleic acid, oleic acid and many fibers. Pumpkin is a powerful antioxidant because of the presence of its phenolic compounds, tocopherols, and zinc. More importantly, in laboratory experiments, there has been an observed significant reduction of cardiotoxicity because of unsaturated fatty acids such as linoleic acid and oleic acid included in the pumpkin that lowers the synthesis of a cyclooxygenase enzyme (Badkoobeh Hezaveh et al. 2021).

#### 2.5.4 Pumpkin Seeds and Pumpkin Seed Oil in Prostate Health

A hypertrophic prostate gland is referred to as benign prostatic hyperplasia. The trials have revealed that pumpkin seeds can reduce BPH symptoms. Pumpkin seeds also improve the health of the bladder. However, it has also been proven that pumpkin seed oil plays an important role in treating benign prostatic hyperplasia. The dosage level of the seed oil is directly proportional to the effect (Arora et al. 2023). Calcium 5‐hydroxytryptophan pumpkin seeds rich in 7‐phytosterols, in particular, decrease the clinical symptoms of benign prostatic hyperplasia (BPH) by inhibiting 5α‐reductase, which reduces the levels of dihydrotestosterone (DHT), thereby reducing prostate growth. In addition, these phytosterols block androgen receptors and prevent even further the growth of prostatic epithelial cells. Clinical studies conducted empirically show that pumpkin seed extracts may produce small benefits in lower urinary tract symptoms, including nocturia and urgency, but they in general do not perform as well as established pharmacotherapies including tamsulosin. Such preparations are in general well‐tolerated and can be a natural supplement or substitute to traditional BPH treatments; however, additional clinical evidence is mandatory before these preparations are approved to make them routinely applied (Kizilirmak et al. 2024).

#### 2.5.5 Hypoglycaemic and Antidiabetic Effects of Pumpkin

Hyperglycemia may result from either decreased response to insulin (DM‐type 2) or the absence of insulin (DMtype 1). The study conducted by (Quanhong et al. 2005) demonstrates that the severe sequelae of chronic hyperglycemia are retinopathy, neuropathy, and nephropathy or pathology of the kidneys, brain, and eyes. The anti‐hyperglycemic properties of Pumpkin that have been proved are in the context of a case–control study carried out in rats. It assists in backing up the information that pumpkin increases the production of insulin and lowers the amount of blood glucose. Pumpkin can help reduce the level of postprandial glucose when it is taken in the first stages of diabetes. The protein‐bound polysaccharide pumpkin (PBPP) has an effect mainly because of the dose‐dependent ability to decrease hyperglycemia in rats. Diabetes mellitus is a long‐term metabolic disorder with several etiological factors. Disturbance of carbohydrate, protein, and lipid metabolism is one of the factors leading to diabetes mellitus. In many other nations, including America, Brazil, Argentina, and India, pumpkins have long been utilized as a diabetes remedy. Pumpkin flour showed a hypoglycemic and hypolipidemic impact, according to a study done on rats with diabetes induced by alloxan. After extensive searching of scientific and research data regarding the effect of the pumpkin on the blood glucose and serum lipids, the current study aimed to define which of the pumpkin parts—peel, flesh, and seeds—produced the strongest potential results of the antihyperglycemic and antihyperlipidemic action in the early phases of manifestations of diabetes in rats that had diabetes induced through the alloxan method (Hussain et al. 2022).

#### 2.5.6 Neuroprotective and Antidepressant Effects of Pumpkin and Pumpkin Seeds

Lipid peroxidation is one of the risk factors in brain damage. The impact of the reaction to generate free radicals, e.g., reactive oxygen species (ROS), is particularly adverse since such radicals damage the cells of the brain. Owing to its high antioxidant capacity, it was also used as the essence of the leaves of the pumpkin plant in a recent study to analyze whether the herb had the potential to keep the brain safe in rats subjected to PEM. The protein located in the seed was visible, and the fluted leaves of the pumpkin closed up to protect the brain cells against PEM‐induced oxidative stress (Potukuchi et al. 2018). Depression is the most common displacement of the brain, which is manifested by the loss of interest in pleasure and sleep eating patterns. They have difficulties focusing and being interested in their day‐to‐day tasks. The number of depressed people worldwide reached 322 million in 2015, per the WHO. Tryptophan concentrations in pumpkin seeds are 576 mg/100 g. This is a neurotransmitter that helps treat depression. The rats were injected with methyl isobutyl ketone in a bid to induce depression. Both raw and processed pumpkin seed extracts have been demonstrated to exert an antidepressant effect (Batool et al. 2022). Researchers have reported the antidepressant properties of pumpkin seeds at 47%. For that reason, pumpkin seeds may have antidepressant qualities. With forced‐swimming and tail‐suspension tests, another study tested the efficacy of the imipramine conventional drug and pumpkin seed extracts in rats. The hypothesis from the study was that pumpkin seed is impregnated with a substantial amount of antidepressant potential (Manshi and Chaturvedi 2023).

#### 2.5.7 Antihypertensive Effects of Pumpkin Seeds and Pumpkin Seed Oil

One of the mechanisms is that pumpkin seeds contain bioactive flavonoids and anthocyanins, which bolster the bioavailability of nitric oxide (NO) known as the critical molecule in blood pressure regulation processes and vasodilation. O relaxes vascular smooth musculature, enhancing the blood flow and lowering resistance. Pumpkin seeds also contain potassium that acts as a natural diuretic and negates the hypertensive action of sodium by promoting sodium excretion and electrolyte balancing (Fajrani et al. 2023). The findings of a study conducted by Andari and Rahayuni (2014) revealed that rats induced to hypercholesterolemia after a period of 14 days significantly reduced the total cholesterol levels of the hypercholesterolemic Wistar rats. In another study, researchers found that the supplementation of pumpkin seed oil is effective in lowering brachial and central blood pressure of postmenopausal women. The regular intake of pumpkin seed oil can reduce heart disease risk (Fajrani et al. 2023).

**FIGURE 7:**
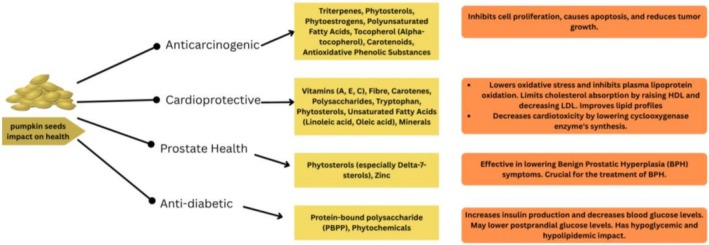
The health impacts of bioactive compounds in pumpkin seeds.

The nutraceutical benefits of pumpkin seeds include anti‐carcinogenic, cardioprotective, prostate health, and hypoglycemic effects, attributable to their rich content of phytochemicals, phytosterols, vitamins, dietary fiber, zinc, magnesium, and PUFAs, as illustrated in Figure 7.

The major bioactive compounds present in Chia, flax, sesame, pumpkin and melon seeds, with their modes of action, are shown in Table 1.

**TABLE 1:** The seeds, their bioactive compounds, and impact on health.

Sr. No.	Seeds	Types of compounds	Bioactive compounds	Mode of action	Impacts	References
1	Sesame, pumpkin, chia, flaxseeds	Phenolic acids	Caffeic acid 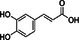	Activates P‐glycoprotein and BCRP and inhibit ER, IGF‐1 receptor, upregulates anti‐apoptotic MCL1, inhibits NF‐κB/STAT3/HIF‐1α, increases the expression of leptin receptor, JAK2, PI3K/Akt, and GLUT3, down‐regulates adiponectin, CEBPA, FABP4 and upregulates CIDEA and UCP1	Anti‐cancer, hepatoprotective, antidiabetic and anti‐obesity	Ayoub et al. (2016); Cecchi et al. (2019); Knez Hrnčič et al. (2019); Pavlíková (2022)
2	Sesame, pumpkin, chia, flax and melon seeds		Gallic acid 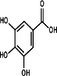	Modulates MAPK/NF‐XB and MyD88 pathways, increase Caspase‐3, Bax, and p53 and decreaseBcl‐2 and p‐AKT, inhinbits IL‐6/STAT3, NF‐KappaB	Anti‐allergy, anti‐obesity, intestinal health, hepatoprotective	Ayoub et al. (2016); Bai et al. (2021); Cecchi et al. (2019); Knez Hrnčič et al. (2019)
3	Chia seeds		Chlorogenic acid 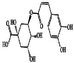	Activates Nrf2/HO‐1 axis, disrupts the TGF‐beta/Smads signaling, increases SOD and GSH, activates NF‐kB, activates Nrf2 antioxidant pathway	Cardioprotective, cerebro‐vascular protection, hypoglecemic	Huang et al. (2023); Sławińska and Olas (2022)
4	Chia seeds		p‐Coumaric acid 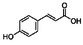	Scavenges ROS, enhances the permeability of the membrane in bacteria and binds to the phosphate anion of DNA, α‐glucosidase, and α‐amylase is inhibited from absorption, resulting	Antioxidant, anti‐cancer, antimicrobial, hypoglycemic, bone health	Ayoub et al. (2016); Kadri et al. (2019); Pei et al. (2016); Sharma et al. (2021); Sławińska and Olas (2022)
5	Chia seeds		Ferulic acid 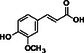	Reduce ROS, stimulate insulin production in rat pancreatic RIN‐5F cells	Antioxidant, anti‐cancer, hypoglycemic	Kadri et al. (2019); Raj and Singh (2022); Sławińska and Olas (2022)
6	Sesame, pumpkin, chia seeds	Flavonoid	Quercetin 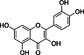	Reacts with glutathione to give non‐reactive metabolites 6‐glutathionyl‐quercetin and 8‐glutathionyl‐quercetin, modulates key signaling pathways (Wnt/beta‐catenin, PI3K/Akt/mTOR, MAPK/ERK, NF‐kappaB, JAK/STAT, Notch)	Anti‐cancer, antioxidant, hypoglycemic, hypoglycemic nephropathy	Ayoub et al. (2016); Michala and Pritsa (2022)
7	Pumpkin, chia seeds		Kaempferol 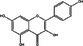	Action on the proliferation of T lymphocytes and normalization of a Th17/Treg, prevents the maturation of dendritic cells, and modulates NF‐κB/mTOR	Intestinal health, immune health, anti‐inflammatory, antioxidant	Ayoub et al. (2016); Chen et al. (2023); Li et al. (2025); Sławińska and Olas (2022)
8	Chia seeds		Rutin 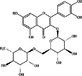	Decreased hypoxia/reoxygenation to cardiomyoblasts TI by stimulating SIRT1 expression, raising levels of SOD, GSH‐Px, CAT, binding to DNA and topoisomerase IIalpha and DNA gyrase B, improved body weight, HbA1c, and inflammatory markers (IL‐6, TNF‐alpha), upregulation of PPAR, and C/EBP‐alpha in 3 T3‐L1 cells	Cardioprotective, anti‐cancer, Neuroprotective, hypoglycemic, anti‐obesity	Semwal et al. (2021); Sławińska and Olas (2022)
9	Chia seeds		Catechins 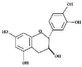	Reduce IL‐1, IL‐6, IL‐12, iNOS, COX‐2, histamine and enhance IL‐4 and IL‐10, modulating the expression of p53, modulate apoptosis‐related protein activity (Bax, Bcl‐2, p53)	Anti‐inflammatory, antioxidant, anti‐atherogenic, anti‐cancer, liver health	Ayoub et al. (2016); Baranwal et al. (2022); Sławińska and Olas (2022); Wang, Gao, et al. (2020)
10	Flaxseeds, chia seeds, sesame seeds, melon seeds, pumpkin seeds	Dietary fiber		Reduce LDL and increase HDL, Fermented to SCFAs generates GLP‐1 and PYY	Cardioprotective, anti‐obesity, gastro‐intestinal health	Barber et al. (2020); Otles and Ozgoz (2025); Shim et al. (2014); Shinde et al. (2023)
11	Flaxseeds, chia seeds	Omega‐3 fatty acids	Alpha‐linolenic acid (ALA) 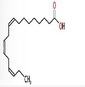	Modulates inflammation and lipid metabolism, helps in the formation of membranes in pericytes and astrocytes, regulates the transport of lipid in the cerebral vessels, manages the blood–brain barrier, and triggers apoptosis in the mitochondria	Cardioprotective, brain health, anti‐cancer, anti‐obesity	Gogna et al. (2022); Imran et al. (2024); Shim et al. (2014); Shinde et al. (2023); Zhu et al. (2024)
12	Flaxseeds	Lignan especially phytoestrogen	Secoisolariciresinol diglycoside (SDG) 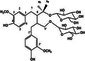	Inhibits NF‐κB, modulates VEGF/IGF‐1, improves the LDL/HDL ratio, liver enzymes (ALT and γ‐GT)	Cardioprotective, bone health, anti‐cancer, liver health	Imran et al. (2015)
13	Sesame seeds	Lignin	Sesamin 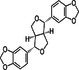	Inhibit NOX2/NOX4, reduce MDA, and increase T‐AOC, to reduce the formation of atherosclerotic lesions in ApoE, prolong aPTT and PT, block the formation of thrombin, 200 mg/day over 8 weeks reduces fasting blood sugar, HbA1c, TNFalpha, IL‐6	Anti‐hypertensive, anti‐atherosclerotic, anti‐thrombotic, hypoglycemic	Andargie et al. (2021); Dalibalta et al. (2020); Jaffar et al. (2025); Meng et al. (2021); Pathak et al. (2018)
14	Sesame seeds		Sesaminol 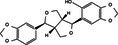	20 μm sesamolin inhibited cell proliferation, migration, and suppress JAK2/STAT3 and MMP‐1, −2, −9 expression, reduces cellular tyrosinase function and tyrosinase, TRP‐1, and TRP‐2 expression, inhibited LDH release and apoptotic cell death in rat PC12 and primary cortical cells induced by hypoxia	Anti‐cancer, anti‐melanogenesis, brain health	Agidew et al. (2021); Andargie et al. (2021); Jaffar et al. (2025); Pathak et al. (2018); Rosalina and Weerapreeyakul (2021)
15	Melon seeds	Squalene	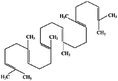	Down‐regulates NF‐kB signaling in neutrophils, and down‐regulates MMPs, it stimulates liver X receptors (LXRs) and PPARalpha and induces the expression of such genes as ABCA1, ABCG1, and ApoE, Modulates NF‐κB/p38 MAPK/HIF	Immune health, hypolipidemic, antioxidant, anti‐cancer	Cheng et al. (2024); Yadav et al. (2025)

## 3 Future Challenges and Perspectives

The future outlook of the nutritional and health potential of edible seeds focuses on harnessing the full potential of the production of sustainable and nutrient‐rich foods. The creation of improved micronutrient bioavailability management by enhancing processing, biofortification and a mechanistic knowledge of micronutrient interactions at the molecular level is an exciting field of research and development. The difficulties connected to the reduction of anti‐nutritional elements, seed mixtures variability, and the guarantee of safety and consumer demand should be tackled. Further, incorporation of edible seeds as functional ingredients in food and nutraceutical sectors would address global malnutrition and chronic diseases by providing bioactive compounds that have antioxidant, anti‐inflammatory and antimicrobial attributes. The sustainability issue should also be emphasized in future research by highlighting why seed by‐products and agricultural waste should be valued as part of the model of a cyclical economy. The solution of these difficulties with the help of multi‐disciplinary methods will guarantee the successful implementation of the edible seeds in the future in terms of human health and nutrition (Sahu et al. 2025).

## 4 Conclusion

The data are clear: seeds are concentrated health assets that can affect key physiological systems. Sesame strengthens the bones and fights oxidative stress, pumpkin protects the prostate and cardiovascular system, chia supports the heart and intestines, flax promotes hormonal and brain health, and melon seeds, often thrown away, offer unrealized antioxidant and anti‐inflammatory effects. They are a wise addition to regular meals because of their nutritional richness and bioactivity, especially in the battle against chronic illnesses. However, achieving their full value requires careful integration, which includes matching them with complementary meals, choosing the right forms (whole, ground, or oil), and regulating portion amounts. The simple seed stands out as a little but powerful weapon for improved nutrition and illness prevention as global health issues increase. There are some potential uses of these seeds. The flaxseed can be used as a substitute for eggs in baking, beneficial for vegans and individuals with egg allergies. These seeds can be used as a healthy option in oatmeal, porridges, cookies, cakes, cupcakes, bread, and energy bars. The study of edible seeds, such as sesame, flaxseed, and pumpkin, as micronutrient sources, considering their bioavailability, health‐promoting effects, and the underlying processes, is hindered by several limitations that need to be mitigated to achieve their maximum potential. The major shortcoming is that concentration and bioavailability of bioactive constituents are heterogeneous and are affected by genetic diversity between cultivars, environmental factors during growth, harvesting timing, and other post‐harvest processes or storage regimes. This heterogeneity is a barrier to the standardization of dosages and the prediction of similar health effects on heterogeneous demographic groups. Limited empirical clinical evidence is available and usually lacks properly powered, controlled studies which are essential in confirming health claims and explaining the chronic effects of seed consumption on human health. Besides, the interaction between constituents of the seeds and the human gastrointestinal microbiota, which is an aspect that has a significant impact on micronutrient uptake and metabolic cleansing, is not studied fully. There are considerable scopes for increasing the bioavailability of vital nutrients using new processing and formulation strategies, such as encapsulation and fermentation, and the aim is to maximize health gains. Furthermore, the economic opportunities of edible seeds in the prevention and control of chronic diseases like cardiovascular disease or diabetes, and individualized nutrition plans are promising approaches in the prevention and control of inflammatory diseases and diabetes. Continued interdisciplinary studies that cut across food science, nutrition, molecular biology, and clinical medicine are essential for the achievement of the maximum potential of such high‐nutrient seeds.

## Author Contributions

**Fakhar Islam:** formal analysis. **Mohd Asif Shah:** software. **Muhammad Afzaal:** data curation.

## Data Availability

All the available data are within the manuscript and further can be taken from the authors on request basis.
